# Whole-brain mapping of projection from mouse lateral septal nucleus

**DOI:** 10.1242/bio.043554

**Published:** 2019-06-17

**Authors:** Ke Deng, Lu Yang, Jing Xie, He Tang, Gui-Sheng Wu, Huai-Rong Luo

**Affiliations:** 1 State Key Laboratory of Phytochemistry and Plant Resources in West China, Kunming Institute of Botany, Chinese Academy of Sciences, Kunming, Yunnan 650201, China; 2 University of Chinese Academy of Sciences, Beijing 100039, China; 3 Key Laboratory for Aging and Regenerative Medicine, Department of Pharmacology, School of Pharmacy, Southwest Medical University, Luzhou, Sichuan 646000, China

**Keywords:** Lateral septal nucleus, H129, PRV, Neural circuits, Neural projection

## Abstract

The lateral septal nucleus (LS) plays a critical role in emotionality, social behavior and feeding processes, through neural connections with the hippocampus and hypothalamus. We investigated the neural circuits of LS by using herpes simplex virus 1 strain H129 (H129) and pseudorabies virus stain Bartha (PRV). Virus H129 indicates that LS directly projects to some cerebral nuclei (nucleus accumbens, bed nuclei of the stria terminalis and amygdala), part of the hypothalamus (median preoptic, paraventricular, dorsomedial nucleus and lateral area), thalamus (medial habenula, the paraventricular, parataenial and reuniens nuclei, and the medial line nuclei) and the pontine central gray. Then the LS has secondary projections to the CA3 and CA1 field of the hippocampal formation, lateral and medial preoptic area, and the mammillary body. PRV tracing shows that LS are mainly receiving primary inputs from the amygdala, hippocampus, hypothalamic, thalamus, midbrain and hindbrain, and secondary inputs from dorsal and central linear nucleus raphe, the lateral part of the superior central nucleus raphe, the ventral anterior-lateral complex, the intermediodorsal nucleus, the central medial nucleus, the rhomboid nucleus, and the submedial nucleus of the thalamus. The neural circuit data revealed here could help to understand and further research on the function of LS.

## Abstract (precis)

**Summary:** We identified the sequence of projections from the lateral septal nucleus by virus tracing and expanded the data on neural circuits, which could help to understand brain function.

## INTRODUCTION

The lateral septal nucleus (LS) is the rostrodorsal part of the septal region, dorsolateral adjacent to the medial septal complex. Usually, the LS is cytoarchitectonically sectioned into dorsal, intermediate and ventral divisions (George and Franklin, 2013). According to Allen Mouse Brain Atlas (Lein et al., 2007), LS in mice consists of caudal, rostral and ventral sections (LSc, LSr and LSv). LS is also surrounded by and shares borders with the nucleus accumbens, lateral preoptic area, bed nuclei of the stria terminalis and fiber tracts such as the medial forebrain bundle system.

Extensive studies have been focused on the functions of LS, such as its critical role in emotionality, social behavior, and feeding processes. The neurons connections in the CA2-LS-ventromedial hypothalamic area can control aggressive behavior (Wong et al., 2016; Leroy et al., 2018). The neural projections from the ventral hippocampus to the LS are a critical component of the anxiety circuit (Parfitt et al., 2017). In the LS, some neurotransmitters such as substance P and serotonin modulate stress response (Gavioli et al., 1999; Ebner et al., 2008); some cell-surface receptors such as vasopressin V1a receptor, metabotropic glutamate receptor 5 and dopamine receptor 3 are involved in complex social behavior (Bielsky et al., 2005; Mesic et al., 2015; Shin et al., 2018); others such as urocortin and endogenous glucagon-like peptide-1 modulate feeding (Wang and Kotz, 2002; Terrill et al., 2018). Also, optogenetics activates the connections between the LS and hypothalamus, which can modulate food intake in mice (Sweeney and Yang, 2015, 2016).

Even though conventional neuroanatomical tracing techniques show the connections of LS with other brain areas (Risold and Swanson, 1997b), they are limited to the analysis of single neurons, not circuits of functionally connected neurons. In general, the LS – along with the septofimbrial and septohippocampal nuclei – sends the vast majority of its projections in a topographically organized way to medial pallidal regions (the medial septal/nucleus of the diagonal band complex and substantia innominate), most of the hypothalamus, parts of the midline thalamus and limited parts of the brainstem. In turn, the LS receives topographically organized input from most of these same regions, and they converge with massive input from the hippocampus, which is also topographically arranged. Research on the complex neural networks of LS has made little progress in the last 20 years.

Neurotropic viruses have been developed as effective trans-neuronal tracers and to analyze central nervous system networks involving chains of two or more functionally connected neurons. The pseudorabies virus (PRV) strain Bartha is an excellent retrograde trans-neuronal tracer, and the herpes simplex virus 1 strain H129 (H129) is an anterograde tracer (Pomeranz et al., 2005; Ekstrand et al., 2008; Jovanovic et al., 2010; Ugolini, 2010; Lo and Anderson, 2011; McGovern et al., 2012; Wojaczynski et al., 2015; Tang et al., 2016b). These viruses replicate themselves in recipient neurons and produce strong trans-neuronal labeling along the projection of neurons; the cascade of projection can also be identified from the replication cycle of the virus (Ekstrand et al., 2008; Ugolini, 2010; Jovanovic et al., 2010; Lo and Anderson, 2011; McGovern et al., 2012).

This study’s purpose was to determine the cascade of projection from the LS. Our results show that a low dose of virus H129 enables the tracing of explicit second-order projection sites in the medial septal nucleus, hypothalamus and hippocampus, and PRV enables the tracing of specific second-order projection sites in the thalamus and raphe nuclei.

## RESULTS

### Virus injection

According to previous reports, the modified H129 and PRV transit the primary-order and second-order neuronal projections at 36–44 h and 52–60 h after injection in mice, respectively (Xie et al., 2018; Tang et al., 2016a). Therefore, in this study, animals were euthanized at 40 and 56 h after virus injection. The injection sites of H129-EGFP virus (5×10^9^ pfu/ml) and PRV-EGFP virus (2×10^10^ pfu/ml) are shown in Figs S1 and S2, respectively. Red fluorescence of cholera toxin subunit B (conjugated Alexa Fluor™ 594) was used in the cocktail to help identify the injection sites. We excluded the unsatisfactory samples, and the injection sites of the remaining animals were highly circumscribed within LSr, specifically around the medial and/or dorsolateral zone of LSr (LSr.m and LSr.dl) as inferred from the subdivision of LSr in rat (Risold and Swanson, 1997a).

### Anterograde projection from the lateral septal nucleus

**Fig. 1.**
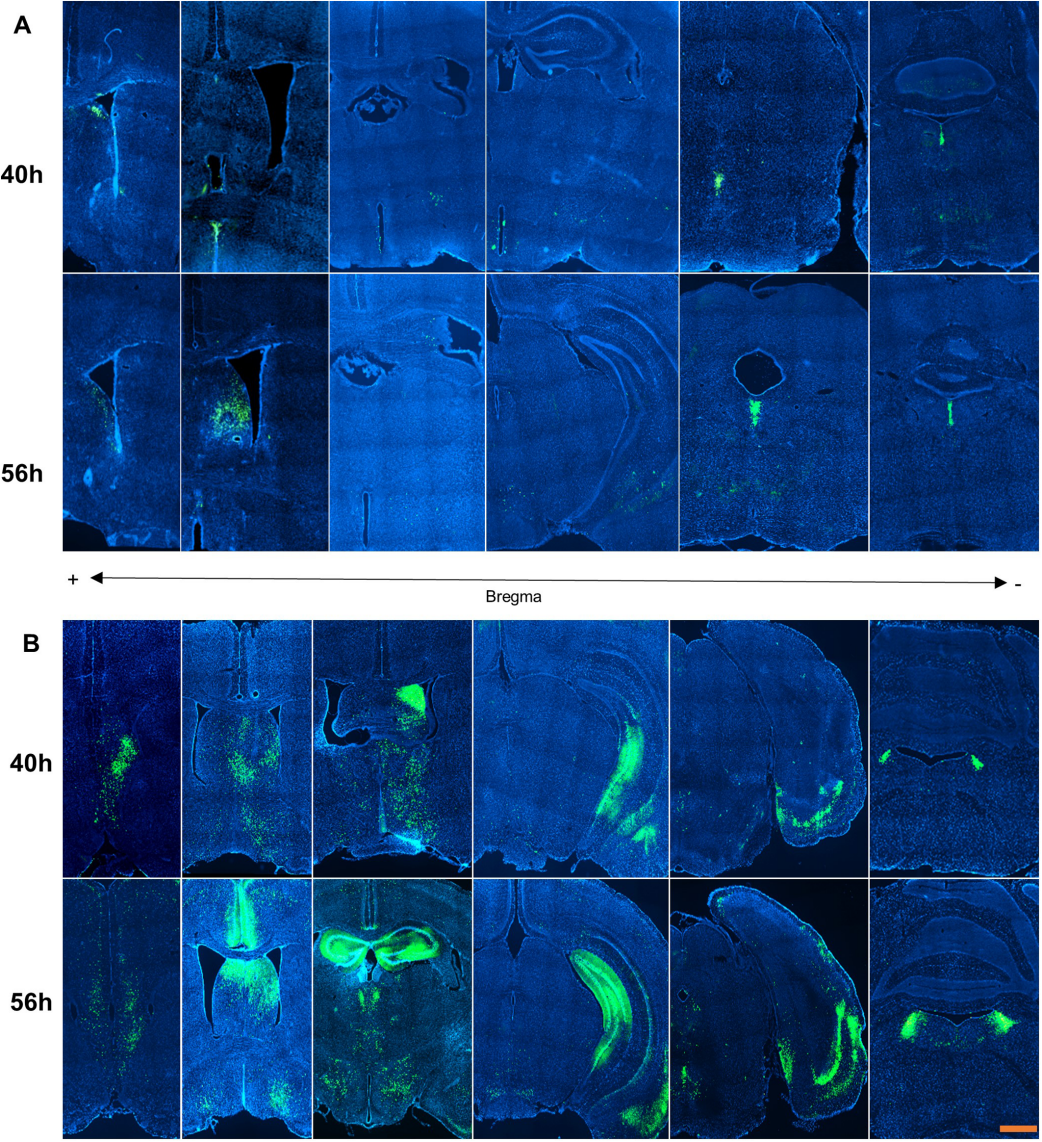
**Overview of the projections from mice rostral part of lateral septal nuclei.** The distribution of projections from mice lateral septal nuclei are summarized. A is the herpes simplex virus 1 strain H129 (HSV-EGFP). B is the pseudorabies virus strain Bartha (PRV-CMV-EGFP). The images in A are reproduced to show the distribution of labeled neurons in more detail in Figs 2, 3 and 5. The images in B are reproduced to show the distribution of labeled neurons in more detail in Fig. 6. Scale bar: 500 μm.

The trace of H129 virus could be identified from the bregma 1.09 mm to −5.51 mm along the rostrocaudal axis 40 h after inoculation, and from the bregma 1.21 mm to −5.63 56 h after inoculation (Fig. 1A, Table 1). For the whole brain, H129 labeled neurons were sparse from the rostral to caudal in the nucleus accumbens, lateral septal complex, medial septal complex, bed nuclei of the stria terminalis, substantia innominate, hypothalamus, medial forebrain bundle system, hippocampal formation, amygdala, thalamus, periaqueductal gray and the pontine central gray.

**Table 1.**
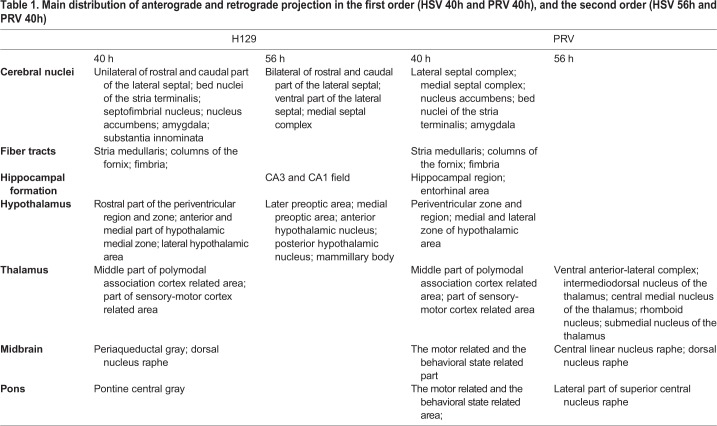
**Main distribution of anterograde and retrograde projection in the first order (HSV 40h and PRV 40h), and the second order (HSV 56h and PRV 40h)**

**Fig. 2.**
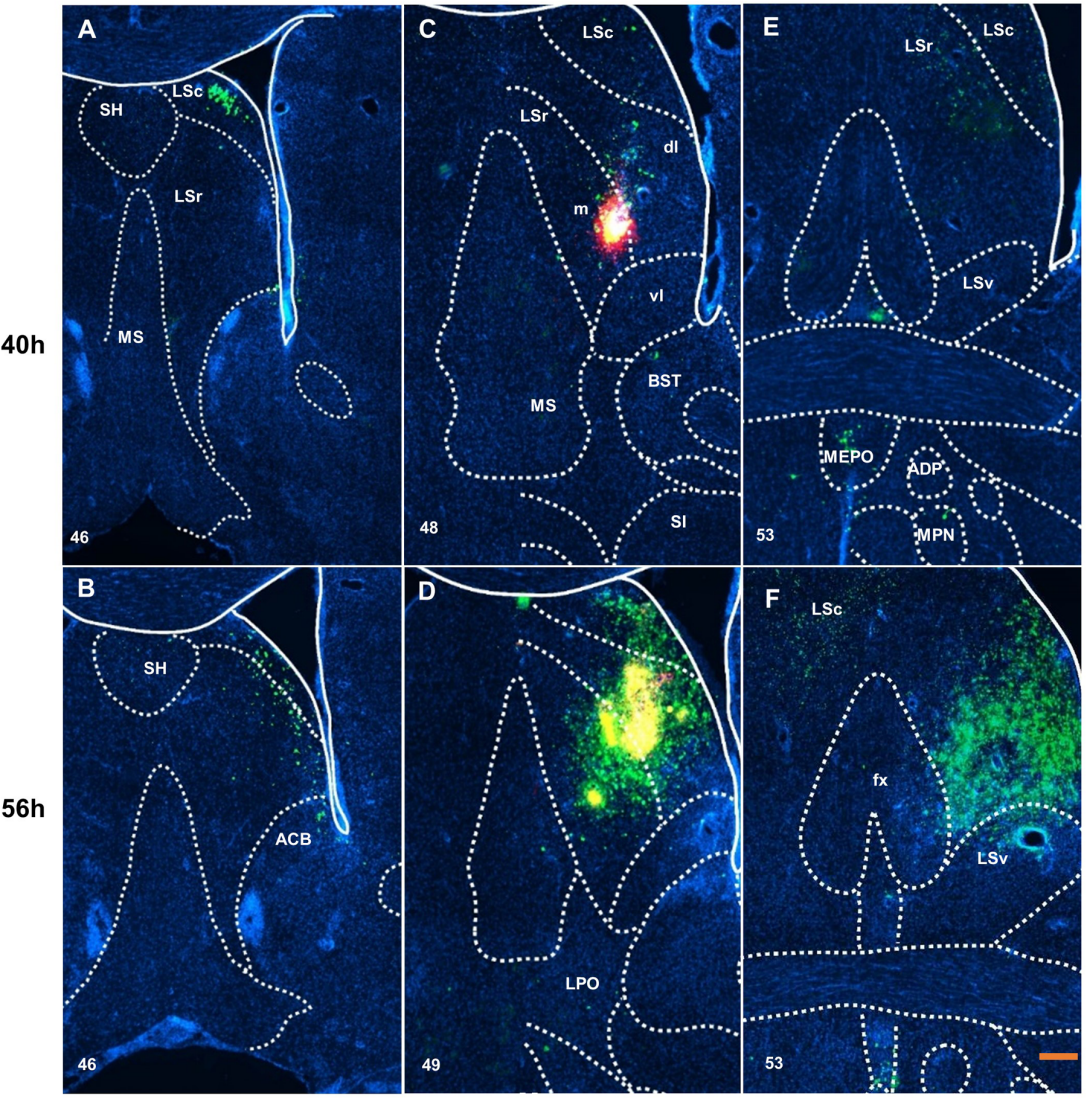
**Distribution of virus H129 labeled neurons around injection sites.** Labeled neurons were mainly scattered in the rostral and caudal part of lateral septal nuclei at 40 h after inoculation (A,C,E) and the bed nuclei of the stria terminalis. Then at 56 h after inoculation (B,D,F), the density of neurons was increased and there was weak input to the medial septal nucleus, lateral and medial preoptic area (D), also to the fornix and the contralateral lateral septal caudal part (F). Scale bar: 100 μm. The numbers in the left corner of the figure panels represent corresponding reference images in the Allen Mouse Brain Atlas. SH, septohippocampal nucleus; LSc, caudal part of the lateral septal nucleus; LSr, rostral part of lateral septal nucleus; MS, medial septal nucleus; dl, dorsolateral zone on LSr; m, medial zone on LSr; vl, ventrolateral zone on LSr; BST, bed nuclei of the stria terminalis; SI, substantia innominata; LSv, ventral part of the lateral septal nucleus; MEPO, median preoptic nucleus; ADP, anterodorsal preoptic nucleus; MPN, medial preoptic nucleus; ACB, nucleus accumbens; LPO, lateral preoptic area; fx, columns of the fornx.

**Fig. 3.**
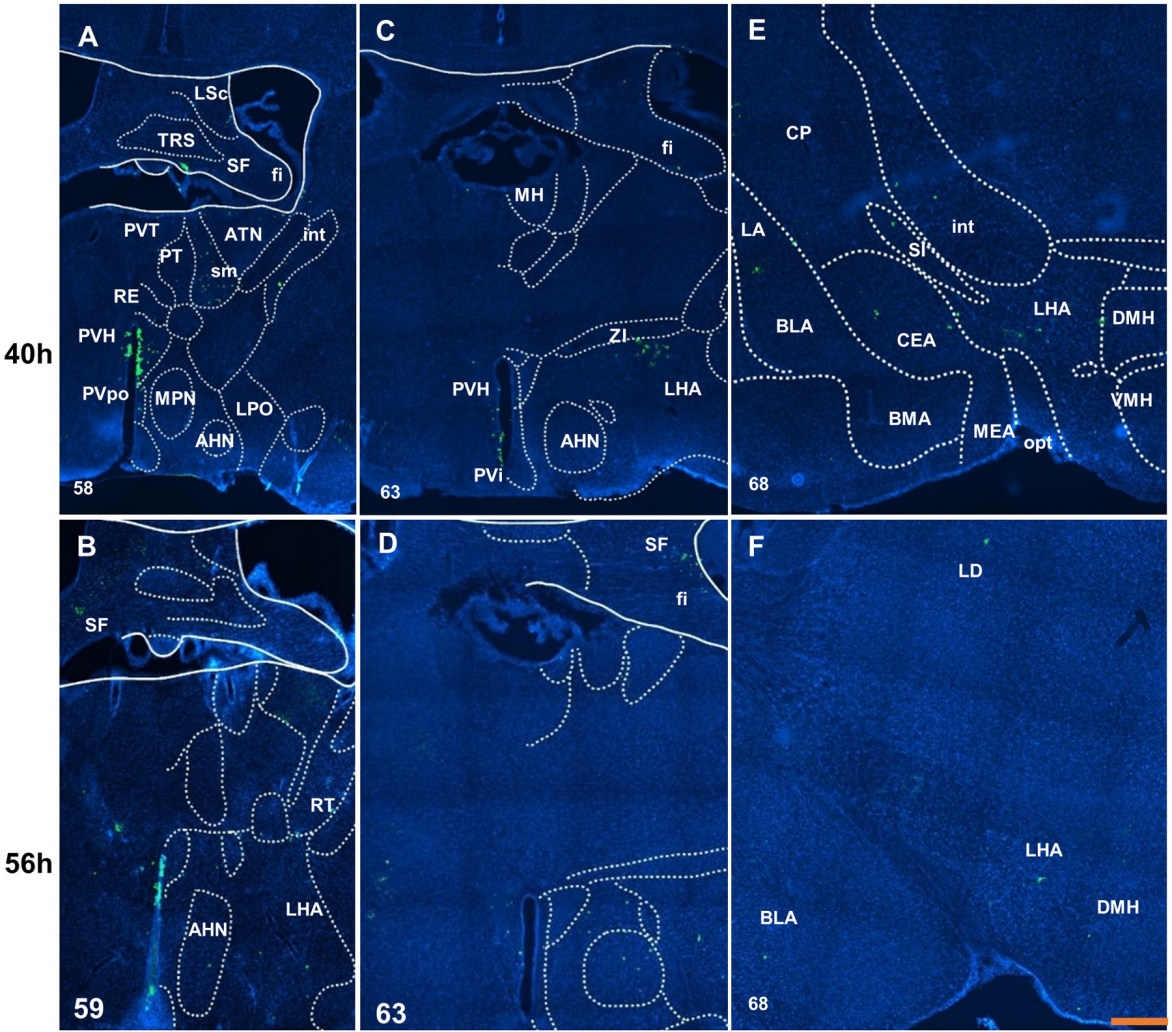
**Distribution of virus H129 labeled neurons in the anterior hypothalamus and amygdala.** Only weak input can be found in the septofimbrial (A for 40 h and B for 56 h) and fimbria (C for 40 h and D for 56 h). The anterior hypothalamic nucleus shows a difference between at 40 h (A,C) and 56 h after inoculation (B,D). The weak and scattered inputs are shown at 40 h after inoculation (E) in basolateral amygdala nucleus and at 56 h after inoculation (F). Scale bar: 250 μm. The numbers in the left corner of the figure panels represent corresponding reference images in the Allen Mouse Brain Atlas. LSc, caudal part of the lateral septal nucleus; TRS, triangular nucleus of septum; SF, septofimbrial nucleus; fi, fimbria; PVT, paraventricular nucleus of the thalamus; ATN, anterior group of the dorsal thalamus; int, internal capsule; PT, parataenial nucleus; sm, stria medullaris; RE, nucleus of reuniens; PVH, paraventricular hypothalamic nucleus; PVpo, preoptic part of the periventricular hypothalamic nucleus; MPN, medial preoptic nucleus; LPO, lateral preoptic area; AHN, anterior hypothalamic nucleus; MH, medial habenula; ZI, zona incerta; LHA, lateral hypothalamic area; PVI, periventricular hypothalamic nucleus, intermediate part; CP, caudoputamen; LA, lateral amygdala nucleus; SI, substantia innominata; DMH, dorsomedial nucleus of the hypothalamus; BLA, basolateral amygdala nucleus; CEA, central amygdala nucleus; BMA, basomedial amygdala nucleus; MEA, medial amygdala nucleus; VMH, ventromedial hypothalamic nucleus; opt, optic tract; RT, reticular nucleus of the thalamus; LD, lateral dorsal nucleus of thalamus.

In lateral septal nucleus, the neurons initially were infected ipsilateral around the injection site, such as in the dorsal zone of LSc (LSc.d) (Fig. 2A), the ventrolateral zone of LSr (LSr.vl) and the bed nuclei of the stria terminalis (BST) (Fig. 2C) within 40 h of inoculation. At 56 h after inoculation, green fluorescence was found in the medial septal complex, the LSv (Fig. 2D) and the contralateral LSc (Fig. 2F). Along the dorsal side of the rostral-caudal axis, weak and scattered projection appeared in the septofimbrial nucleus and the fimbria (Fig. 3A,C) at 40 h after inoculation. At 56 h after inoculation, labeled neurons could be identified at the columns of the fornix (Fig. 2F).

**Fig. 4.**
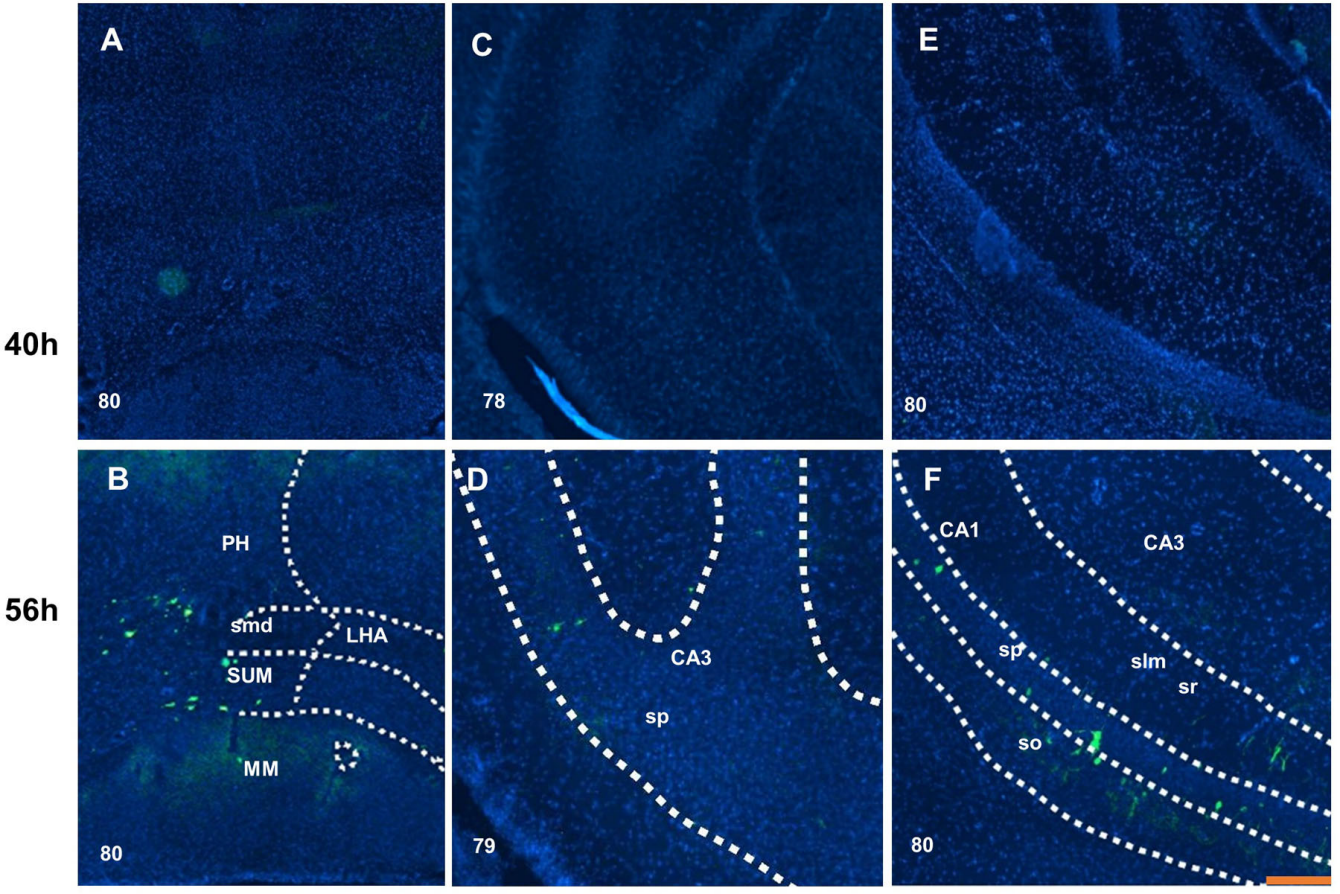
**Distribution of virus H129-labeled neurons in the posterior hypothalamus and the ventral hippocampus.** The posterior hypothalamic nucleus, supramammillary nucleus and mammillary nucleus received second-order projection at 56 h after inoculation, but not at 40 h (B). Some labeled neurons showed up on the pyramidal layer of CA3 field at 56 h (D) but not at 40 h after inoculation (C). In the field of CA1, moderate input was distributed from the stratum oriens to the pyramidal layer, stratum radiatum and stratum lacunosum-molecular (F) at 56 h but not at 40 h after inoculation (E). Scale bar: 200 μm. The numbers in the left corner of the figure panels represent corresponding reference images in the Allen Mouse Brain Atlas. PH, posterior hypothalamic nucleus; smd, supramammillary decussation; LHA, lateral hypothalamic area; SUM, the supramammillary nucleus; MM, medial mammillary nucleus; CA3, field CA3; sp, pyramidal layer; CA1, field CA1; slm, stratum lacunosum-molecular; sr, stratum radiatum; so, stratum oriens.

In the amygdala and adjacent areas (striatum-like amygdala nuclei and lateral, basolateral and basomedial amygdala nucleus), the projection appeared in the lateral amygdala nucleus, basolateral amygdala nucleus, central amygdala nucleus and substantia innominate at 40 h after inoculation (Fig. 3E). In the hypothalamus at 40 h after inoculation, the periventricular zone and periventricular region show moderate input, especially in the median preoptic area (Fig. 2E), paraventricular hypothalamic nucleus (Fig. 3A) and few in the anterodorsal preoptic nucleus (Fig. 2E). However, scattered input emerged in the hypothalamic lateral and medial zone, such as in the medial preoptic nucleus (Fig. 2E), lateral hypothalamic area (Fig. 3C and E), zona incerta (Fig. 3C) and dorsomedial nucleus of the hypothalamus (Fig. 3E). At 56 h after inoculation, infected neurons could be found at the medial preoptic area, the lateral preoptic area (Fig. 2D), the anterior hypothalamic nucleus (Fig. 3B,D), the posterior hypothalamic nucleus, the supramammillary nucleus and the medial mammillary nucleus (Fig. 4B).

At 40 h after inoculation in the thalamus, the labeled neurons could be found in the polymodal association cortex related area, including the paraventricular nucleus, the parataenial nucleus, the nucleus of reuniens, the anterior group of the dorsal thalamus (Fig. 3A) and medial habenula (Fig. 3C). The stria medullaris, which belongs to the medial forebrain bundle system, also shows an obvious signal (Fig. 3A). No noticeable change was found at 56 h after inoculation.

**Fig. 5.**
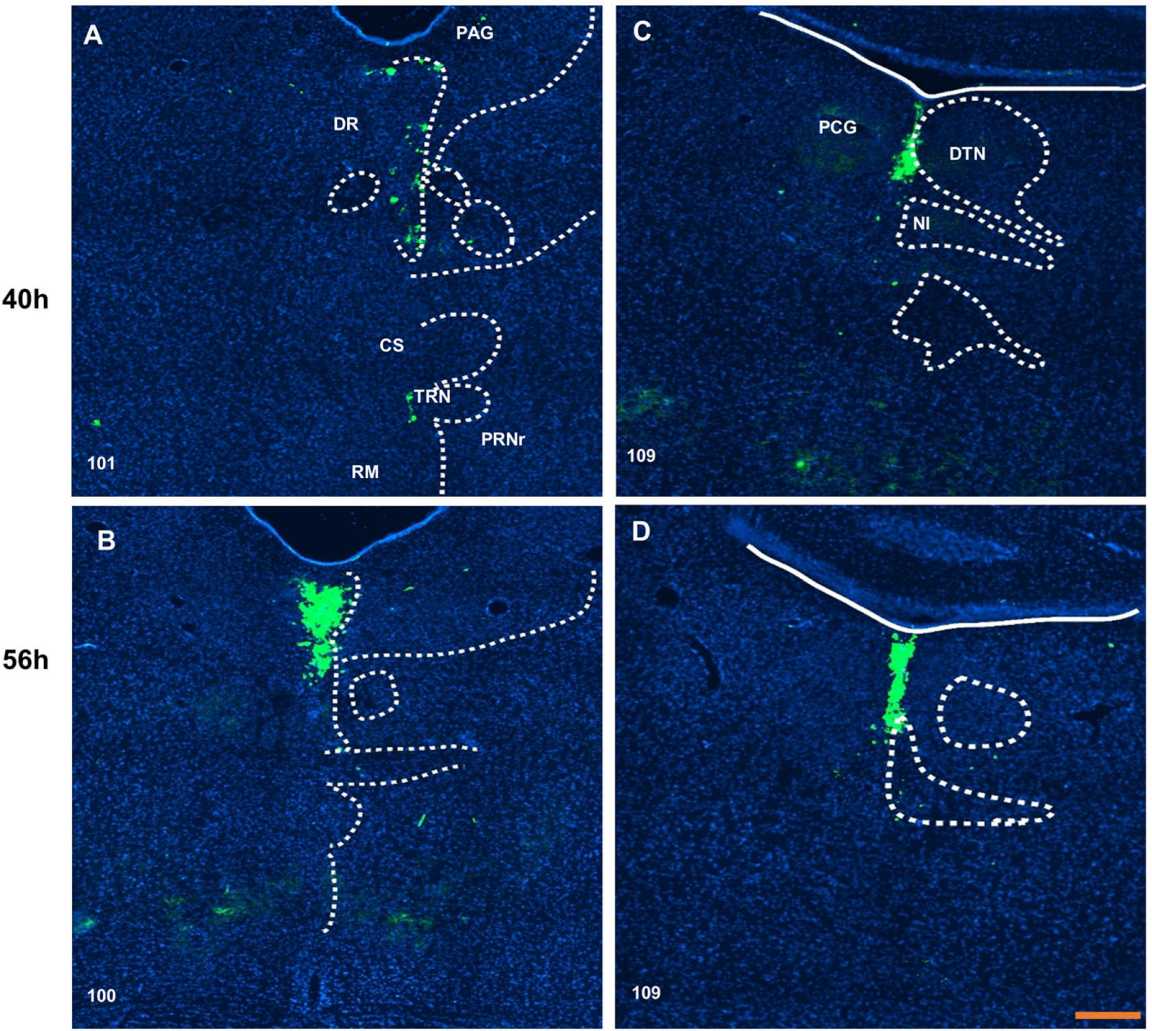
**Distribution of virus H129-labeled neurons in the midbrain and pontine.** The labeled neurons in dorsal nucleus raphe (A,B) and pontine central gray (C,D) show density change over time. Scale bar: 200 μm. The numbers in the left corner of the figure panels represent corresponding reference images in the Allen Mouse Brain Atlas. PAG, periaqueductal gray; DR, dorsal nucleus raphe; CS, superior central nucleus raphe; TRN, tegmental reticular nucleus; PRNr, pontine reticular nucleus; RM, nucleus raphe magnus; PCG, pontine central gray; DTN, dorsal tegmental nucleus; NI, nucleus incertus.

In the hippocampal area, no trace of infection could be found at 40 h after inoculation. At 56 h after injection, the field of CA3 received input in the pyramidal layer (Fig. 4D). Moreover, the field of CA1 also had labeled neurons in the pyramidal layer; other neurons and fibers could be found in the stratum oriens, stratum radiatum and stratum lacunosum-molecular (Fig. 4F). Midbrain, periaqueductal gray and dorsal nucleus raphe showed a weak signal at 40 h after inoculation (Fig. 5A); however, the infected neurons were more abundant at 56 h after inoculation (Fig. 5B).

The middle of pontine central gray (Fig. 5C,D), which is ventral to the fourth ventricle, dorsal to the nucleus incertus and between the dorsal tegmental nucleus, showed traces of infection at 40 h after inoculation. Over time the density of infected neurons increased in this area.

### Retrograde tracer PRV shows projections to the lateral septal nucleus

At 40 h after inoculation, the distribution of PRV could be found in similar areas infected with H129 virus, with the field of CA2 and the entorhinal area in hippocampal formation, and the parvicellular reticular nucleus labeled by PRV only (Fig. 1B).

The site of PRV injection was also identified within the LSr.m and LSr.dl (Fig. S2). At 40 h after virus injection, the trace of infection was found at the lateral septal complex (LSc, LSr and septohippocampal nucleus) and medial septal complex (medial septa and diagonal band nucleus), nucleus accumbens and bed nuclei of the stria terminalis (Fig. S3A,C,E); the dorsal part of taenia tecta (Fig. S3A), substantia innominata (Fig. S3E,G) and magnocellular nucleus (Fig. S3G).

In the rostral part of the hypothalamic area at 40 h after virus injection (Fig. S3E,G,I), periventricular zone (such as paraventricular hypothalamic nucleus), periventricular region (include median and medial preoptic area, anterodorsal preoptic nucleus, anteroventral periventricular nucleus, anteroventral preoptic nucleus and ventrolateral preoptic nucleus) and adjacent areas such as lateral preoptic area, were infected by PRV. In the hypothalamic medial zone and adjacent area, the medial preoptic nucleus (Fig. S3I,K) and anterior and ventromedial hypothalamic nucleus (VMH) (Fig. S3M) had labeled neurons. Nearby, scattered labeled neurons were distributed in the amygdala (Fig. S3M,O).

**Fig. 6.**
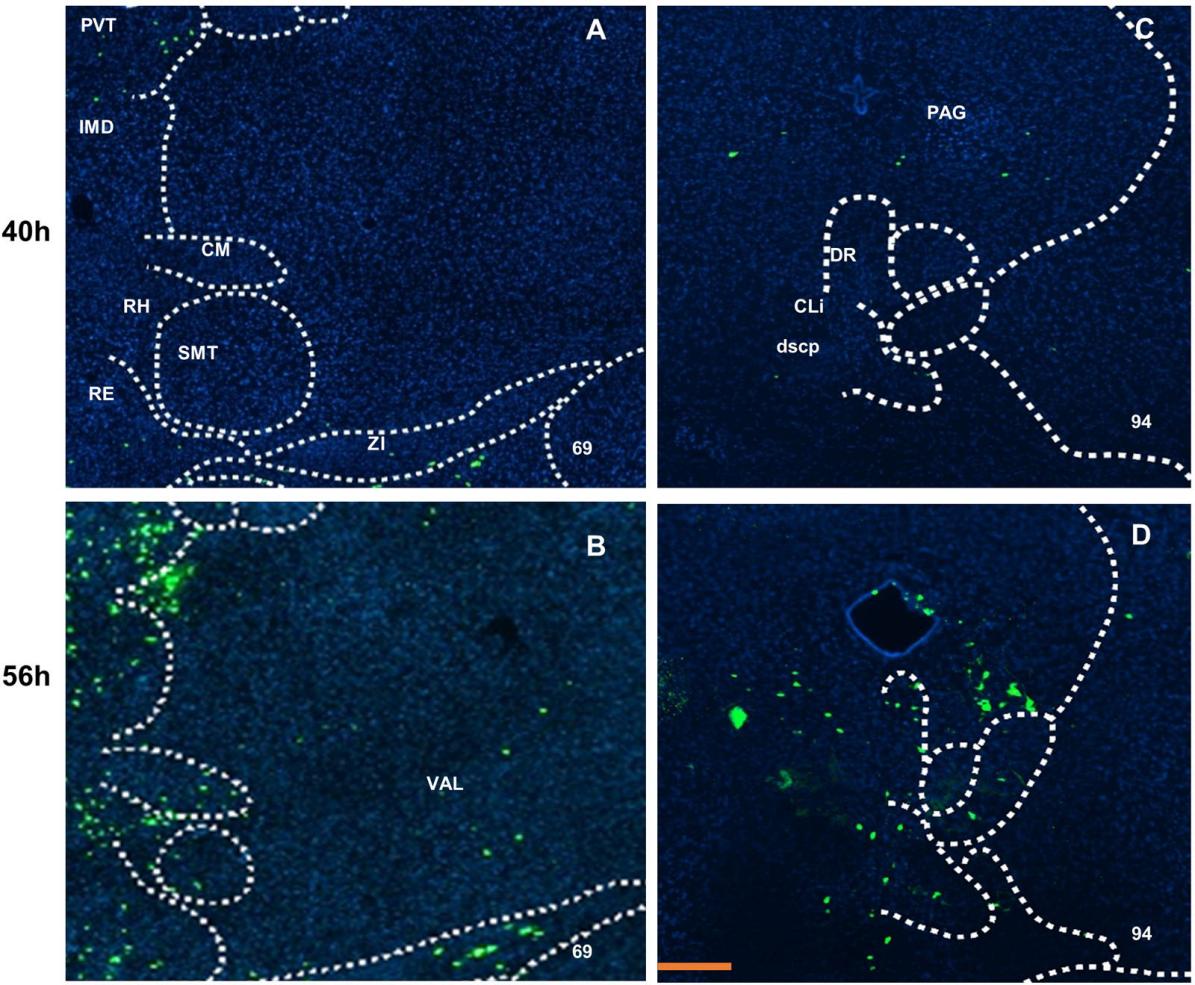
**Projection of virus PRV-labeled neurons in the thalamus and midbrain.** The density of labeled neurons shown in the intermediodorsal nucleus, central medial nucleus, submedial nucleus and rhomboid nucleus of the thalamus. The ventral anterior-lateral complex of the thalamus received signals at 56 h after inoculation (B), the central linear nucleus raphe also had input at 56 h after inoculation (D), but not at 40 h after inoculation (A,C). Scale bar: 200 μm. The numbers in the left corner of the figure panels represent corresponding reference images in the Allen Mouse Brain Atlas. PVT, paraventricular nucleus of the thalamus; IMD, intermediodorsal nucleus of the thalamus; CM, central medial nucleus of the thalamus; RH, rhomboid nucleus; SMT, submedial nucleus of the thalamus; RE, nucleus of reuniens; ZI, zona incerta; PAG, periaqueductal gray; DR, dorsal nucleus raphe; CLi, central linear nucleus raphe; dscp, superior cerebellar peduncle decussation; VAL, ventral anterior-lateral complex of the thalamus.

At 40 h after virus injection in the thalamus areas, such as the paraventricular nucleus, the nucleus of reuniens, and medial habenula (Fig. S3K,M,O), disperse labeled neurons were identified. In addition, labeled neurons were found in the stria medullaris and stria terminalis (Fig. S3K,M). At 56 h after inoculation, labeled neurons in the ventral anterior-lateral complex, the mediodorsal nucleus, central medial nucleus, rhomboid nucleus and submedial nucleus of the thalamus could be found (Fig. 6B; Fig. S3P).

The parts of hippocampus, such as field CA1, CA2, CA3, the dentate gyrus, the ventral part of the subiculum, the lateral part of the entorhinal area (Fig. S3M,O,Q,S) and the amygdala (including the medial amygdala nucleus, central, basomedial and lateral amygdala nucleus) (Fig. S3K,M,O,P,Q,S) also have labeled neurons at 40 h after virus injection.

In the midbrain, the motor related part, including the periaqueductal gray, the intermediate gray layer of the motor-related superior colliculus, the midbrain reticular nucleus and the retrorubral area of midbrain reticular nucleus have scattered labelled neurons and a weak signal at 40 h after virus injection (Fig. S3Q,S).

In the hindbrain, the motor-related area, including the tegmental reticular nucleus and pontine central gray, in addition to the behavioral-state related pons area, including the pontine reticular nucleus, the medial part of the superior central nucleus raphe, locus ceruleus and nucleus incertus (Fig. S3U,W), also have labeled neurons.

Also, the dorsal nucleus raphe and central linear nucleus raphe in the midbrain (Fig. 6D; Fig. S3T), and in the pons, the lateral part of the superior central nucleus raphe, received input at 56 h after virus injection (Fig. S3T).

## DISCUSSION

We investigated the whole-brain map of neuronal projections to and from LS by injected H129 or PRV to the LSr. Both anterograde trans-neuronal virus H129 and retrograde spreading virus PRV tracing show particular trans-regional projection of LS.

Virus H129 indicates LSr directly projects to: (1) some cerebral nuclei, including the nucleus accumbens, bed nuclei of the stria terminalis and the amygdala; (2) part of the hypothalamus, such as the median preoptic nucleus area, the paraventricular hypothalamic nucleus, the lateral hypothalamic area and the dorsomedial nucleus of the hypothalamus. (3) Part of the thalamus, containing the paraventricular nucleus, the parataenial nucleus, the nucleus of reuniens, the medial habenula, and midline nuclei; (4) the pontine central gray. Then secondarily projects to the LSv, the medial septal nucleus, the field CA3 and the field CA1 of the hippocampal formation, lateral and medial preoptic area, and the mammillary body.

PRV tracing shows LS receive many more direct projections from the above area. Then secondary projection from several raphe (dorsal nucleus raphe and central linear nucleus raphe), the lateral part of superior central nucleus raphe in the pons, and part of the thalamus (the ventral anterior-lateral complex, the intermediodorsal nucleus, the central medial nucleus, the rhomboid nucleus and the submedial nucleus).

**Fig. 7.**
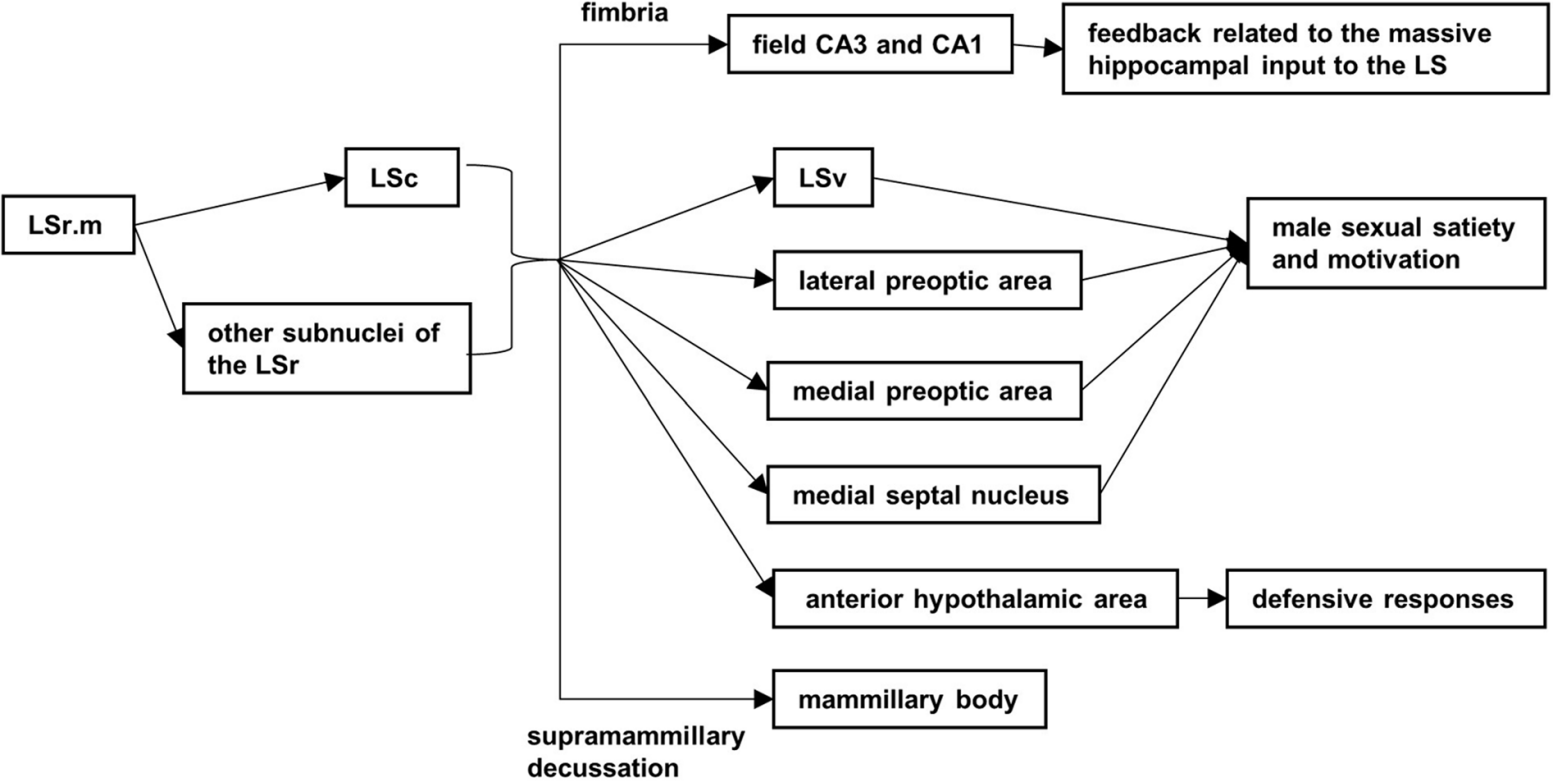
**The possible anterograde secondary projections and relation functions.**

Our results suggest that the secondary projections cross through medial brain fiber tracts (such as the fimbria) to reach the field CA3 and CA1, and traveling along the supramammillary decussation to the mammillary body, might come from the subsequent projection of subnucleus in the LS (Fig. 7). The former projection is usually thought of as feedback related to the massive hippocampal input to the LS (Staiger and Nurnberger, 1991b).

As the dorsal subnucleus of the lateral septal nucleus sent the largest number efferent fibers to the medial septal nucleus than other subnuclei (Staiger and Nurnberger, 1991a), that may result in the LSc (or other subnuclei of the LSr) projected to the LSv, the medial septal nucleus, the lateral and medial preoptic area and the anterior hypothalamic area at 56 h after infection (Fig. 7). Those projections may be involved in male sexual satiety and motivation (He et al., 2013; Gulia et al., 2018), and defense response (Hakvoort Schwerdtfeger and Menard, 2008; Chee and Menard, 2011; Lamontagne et al., 2016).

Retrograde tracing results were more complicated at the nucleus accumbens, some nuclei in the amygdala and hypothalamus may provide mainly secondary projection to the thalamus and raphe nuclei, those projections may be involved in emotion, cognitive and sensorimotor processes (see Van der Werf et al., 2002 for a review).

Even though both virus tracers have been tested in previous research (Xie et al., 2018; Tang et al., 2016a), some nonspecific spread through the cerebrospinal fluid and uptake by fibers of passage needs to be considered, such as the idea that the dorsal nucleus of raphe could be nonspecifically labeled by Bartha PRV (Chen et al., 1999). Avoiding any leak of the virus during the injection process is helpful to get a better result. The density of retrogressive labeled neurons in the locus ceruleus and dorsal nucleus of raphe at 40 and 56 h after inoculation suggested that nonspecific spread was not evident in our results.

The identification of neural networks is fundamental to understanding brain function. Here we showed that there is a clear order of projection in the foundation of the functional process of LS. These revealed neural circuit data that should guide further research in the function of LS and underlying behavior mechanisms. Future characterization of the neurotransmitters and other signaling molecules, receptors and ion channels of the neurons involved in these neural circuits would improve our understanding of the functional mechanism of LS.

## MATERIALS AND METHODS

### Animals

The method used in this article is similar to our previous research (Tang et al., 2016a; Xie et al., 2018). In brief, C57BL/6 male mice (6 weeks, 18–22 g) purchased from Vital River Co., Ltd. (Beijing, China) were housed four per cage under pathogen-free conditions with a 12/12 h light/dark cycle, temperature of 22±2°C, relative humidity 50–60%, with free access to food and water. Animals were habituated to the facility environment for 1 week before the surgical procedure. All experiments were conducted according to the EC Directive 86/609/EEC for animal experimentation, and the experimental procedures were approved by the Animal Care and Use Committee of Wuhan Institute of Physics and Mathematics and the Kunming Institute of Botany, Chinese Academy of Sciences.

### Virus injection

The herpes simplex virus 1 strain H129 (H01001 HSV-EGFP) and pseudorabies virus strain Bartha (P01001 PRV-CMV-EGFP) were supplied by BrainVTA Co. Ltd (Wuhan, China). 100 nl of cocktail containing 60 nl HSV-EGFP (5×10^9^ pfu/ml) and 40 nl 0.05% recombinant cholera toxin subunit B conjugated Alexa Fluor™ 594 (CT-B, Thermo Fisher Scientific, C22842) or cocktail containing PRV-CMV-EGFP (2×10^10^ pfu/ml) and 40 nl 0.05% cholera toxin subunit B was injected into the medial zone on rostral lateral septal nucleus with coordinate AP/DV/ML: 0.6/0.3/3.3 mm using a pulled glass pipette on a speed of 30 nl/min (Nanoliter 2000, WPI, USA). After the injection, the pipette was left in place for 15 min and then was drawn out gently. Four mice at 40 h and four mice at 56 h were euthanized. All operations with the virus were conducted in a Level-2 Biosafety laboratory.

### Histology and immunostaining

The histology and immunostaining were conducted as previously described (Xie et al., 2018; Tang et al., 2016a). After pentobarbital overdose, mice were intracardially perfused with 0.9% saline solution followed by 4% paraformaldehyde in PBS. Mouse brains were removed immediately and placed in 4% paraformaldehyde solution overnight for post-fixation and then soaked in 30% sucrose solution for at least 48 h for cryoprotection. With a cryostat (Leica CM1950), the brains were sequentially cut into 40-μm-thick coronal sections from the olfactory bulb to the cerebellum (approximately +4.50 mm to −6.40 mm from the bregma). The sections were cleaned with PBS and mounted on chrome-gelatin subbed glass slides in sequence. To enhance the fluorescence intensity of the HSV-EGFP labeled slice, the sections were blocked with 3% BSA in PBS-0.3% Triton X-100 for 1 h at 37°C and subsequently incubated with anti-GFP antibody conjugation FITC (1:400, Abcam, ab6662) for 2 h at 37°C. After washing, brain sections were coverslipped with 70% DAPI-glycerol mounting medium.

### Photograph and analysis

The images of whole-brain sections were acquired with a digital slide scanner (Nikon Eclipse Ti) and adjusted by NIS-Elements. The locations of the labeled neurons and outlines of the brain nuclei were manually defined according to the Allen Mouse Brain Atlas (Lein et al., 2007), and the subdivision of the lateral septal nucleus in rats (Risold and Swanson, 1997a).

## Supplementary Material

Supplementary information
