# Supplementary material for: Whole-brain mapping of projection from mouse lateral septal nucleus
Source: Biol Open. 2019 Jun 17;8(7):bio043554. doi: 10.1242/bio.043554 (PMC6679409; doi:10.1242/bio.043554)
Supplement: Supplementary information [file biolopen-8-043554-s1.pdf]

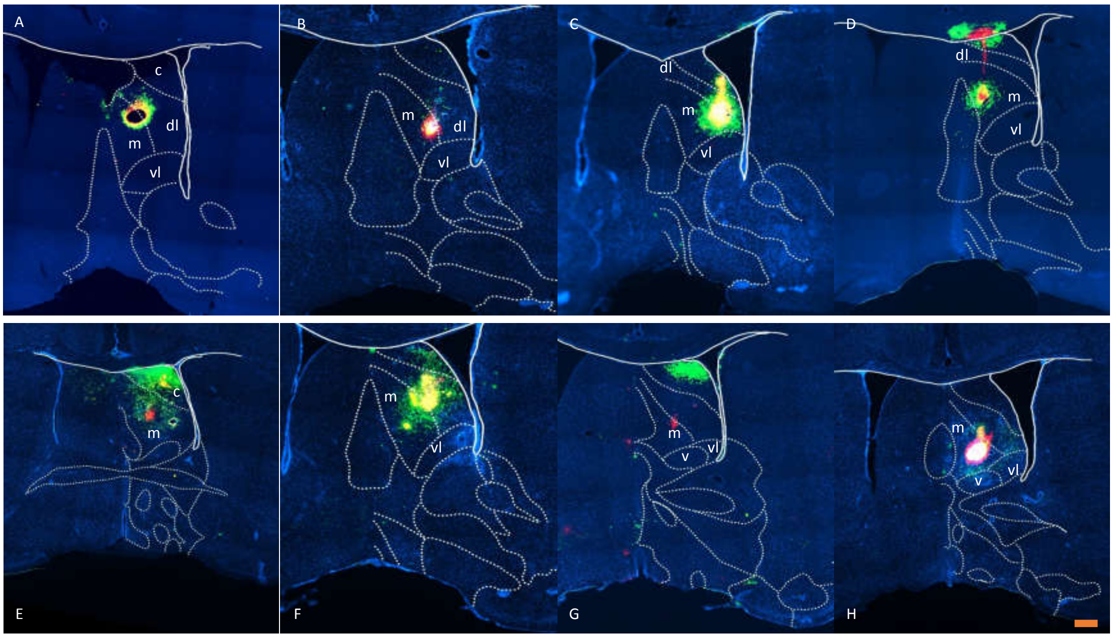

Fig. S1. Injection sites of virus H129-EGFP (green) and CTB (red). The anterograde projection was labeled by HSV, A, B, C, and D were 40 hours sacrificed mouse, E, F, G, and H were 56 hours sacrificed mouse. Injection sites were centered in the boundary of medial and dorsolateral zones in experiments HSV40h1 (A), HSV40h2 (B), HSV40h3 (C) and HSV56h2 (F), and in the medial zones in experiments HSV56h1 (E) and HSV56h4 (H). Some leak was happened in experiment HSV40h4 (D) around the genu of corpus callosum and in experiment HSV56h3 (G) around the caudal part of lateral septal. The orange bar was present 250μm.

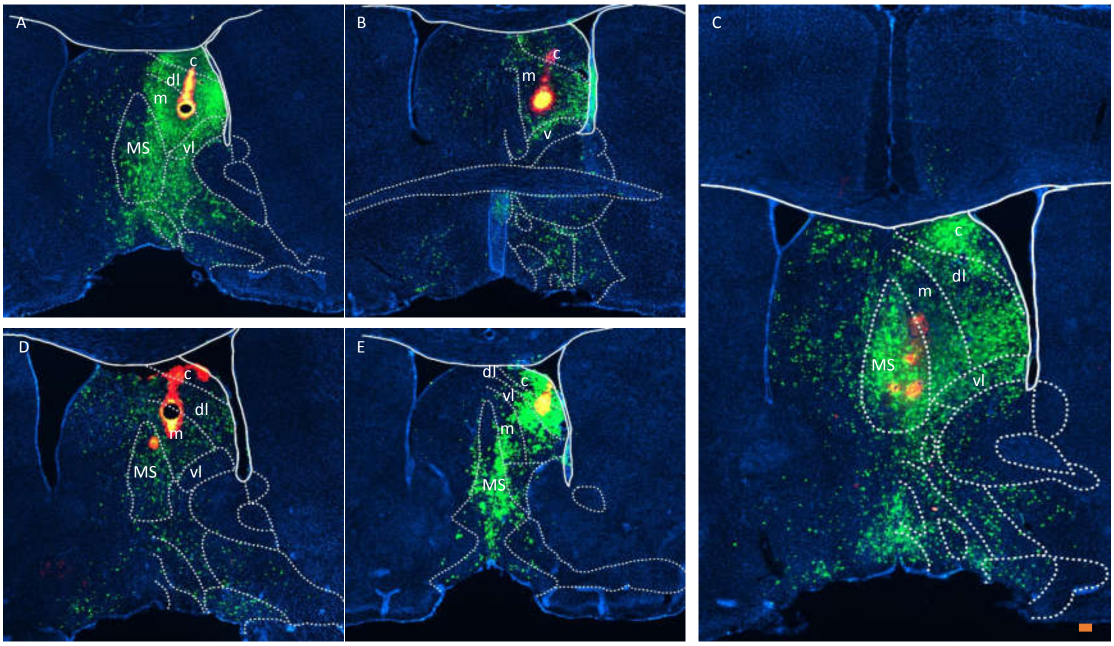

Fig. S2. Injection sites of virus PRV-CMV-EGFP (green) and CTB (red).

The PRV-labeled retrogressive projection, A, B, and C were 40 hours sacrificed mouse, D and E were 56 hours sacrificed mouse. Injection sites were almost same in experiments PRV40h1 (A) and PRV56h1 (D), in the boundary of medial and dorsolateral zones, whereas in experiment PRV40h2 (B) more center in medial zone, and in experiment PRV40h3 (C) adjacent medial septal nucleus and in experiment PRV56h2 (E) in the dorsolateral zone as encroach on caudal part of later septal nucleus. The orange bar was present 250μm.

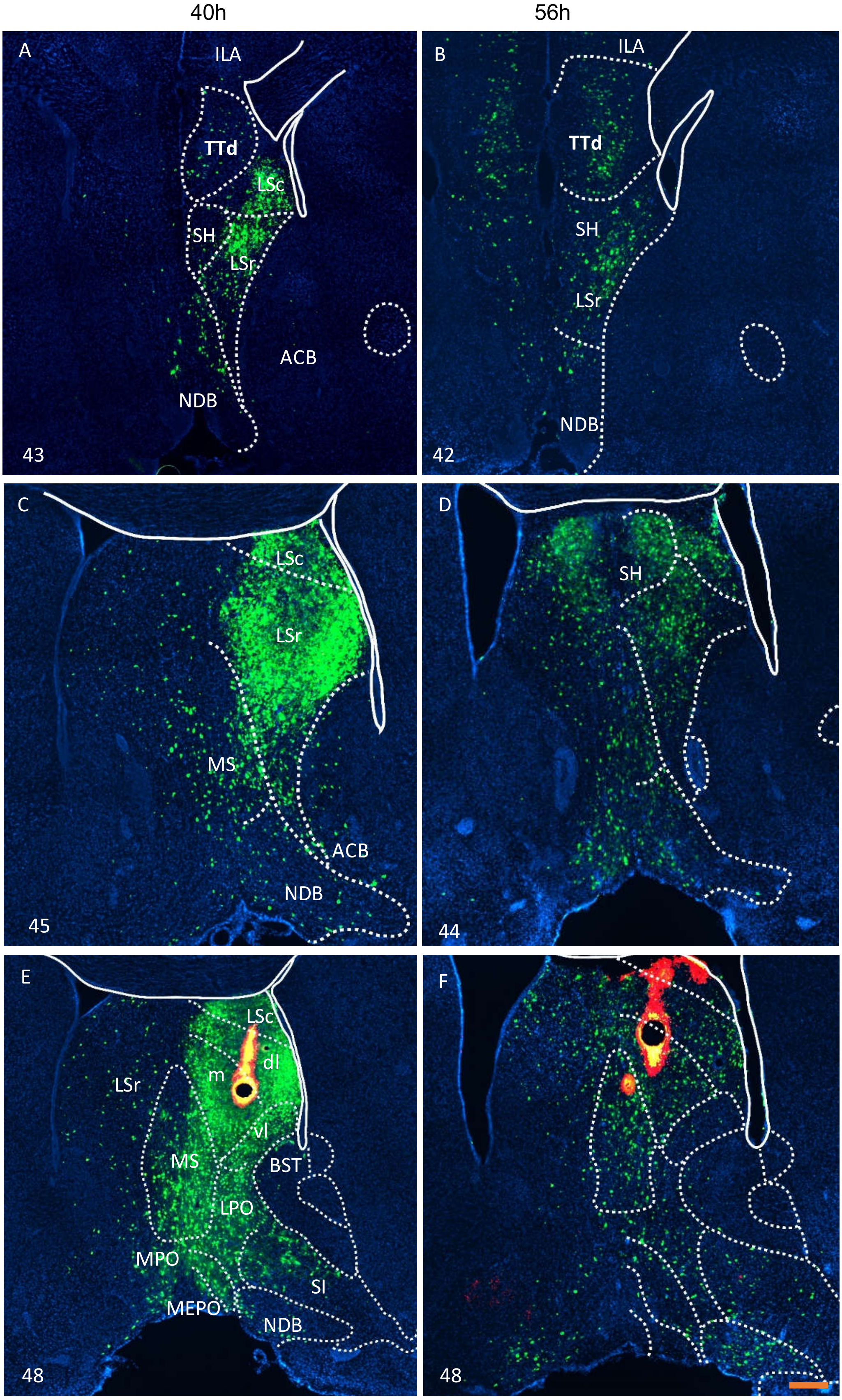

Biology Open • Supplementary information

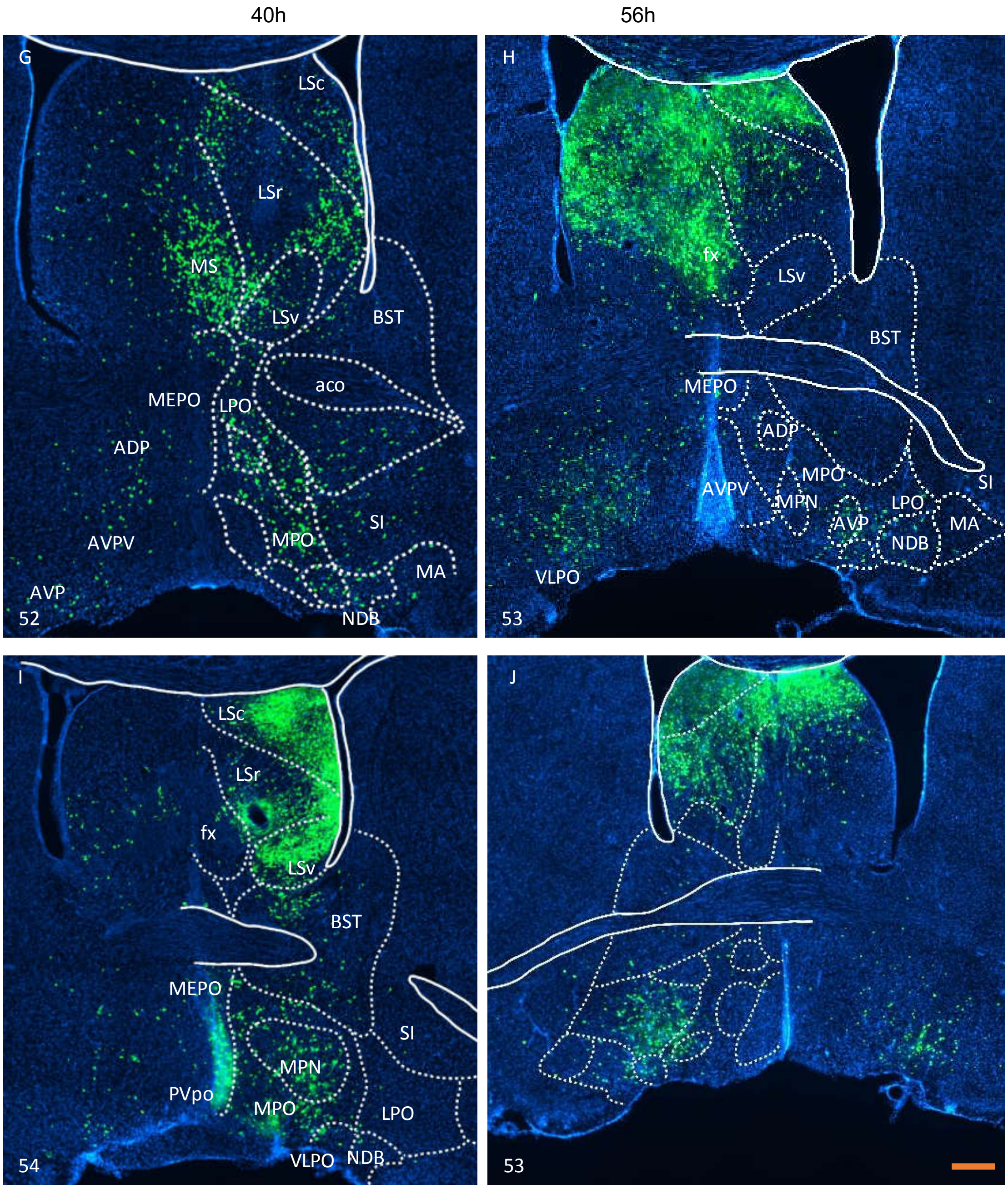

40h
56h
G
LSc
LSr
MS
LSv
BST
aco
MEPO
LPO
ADP
AVPV
MPO
SI
MA
AVP
52
NDB
H
fx
LSv
BST
MEPO
ADP
MPO
SI
AVPV
MPN
LPO
AVP
MA
NDB
VLPO
53
I
LSc
LSr
fx
LSv
BST
MEPO
SI
MPN
PVpo
MPO
LPO
54
VLPO
NDB
J
53

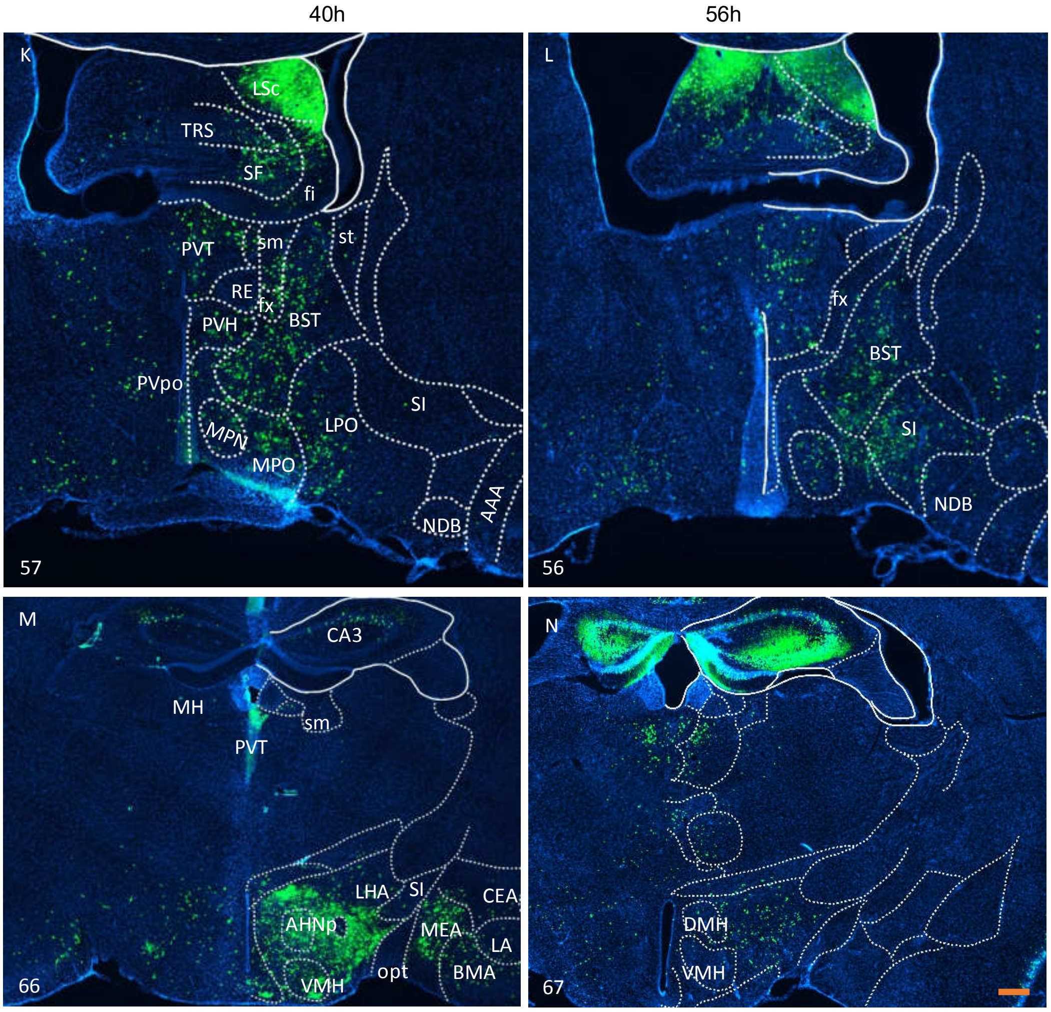

Biology Open • Supplementary information

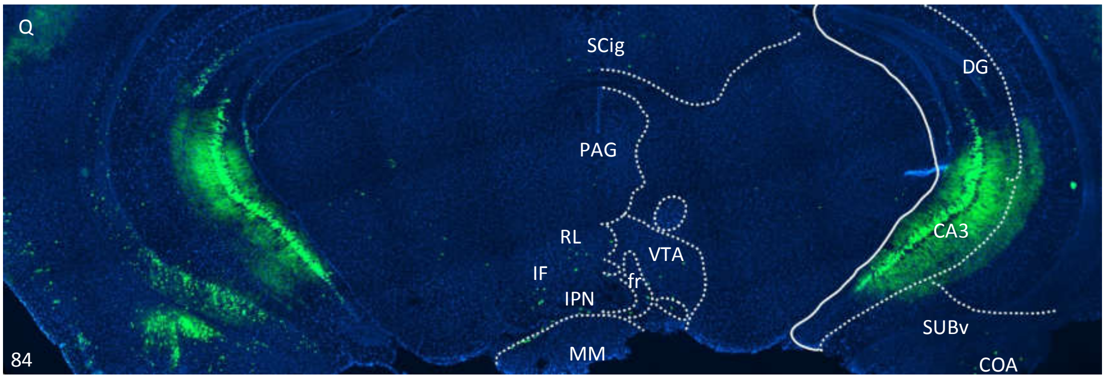

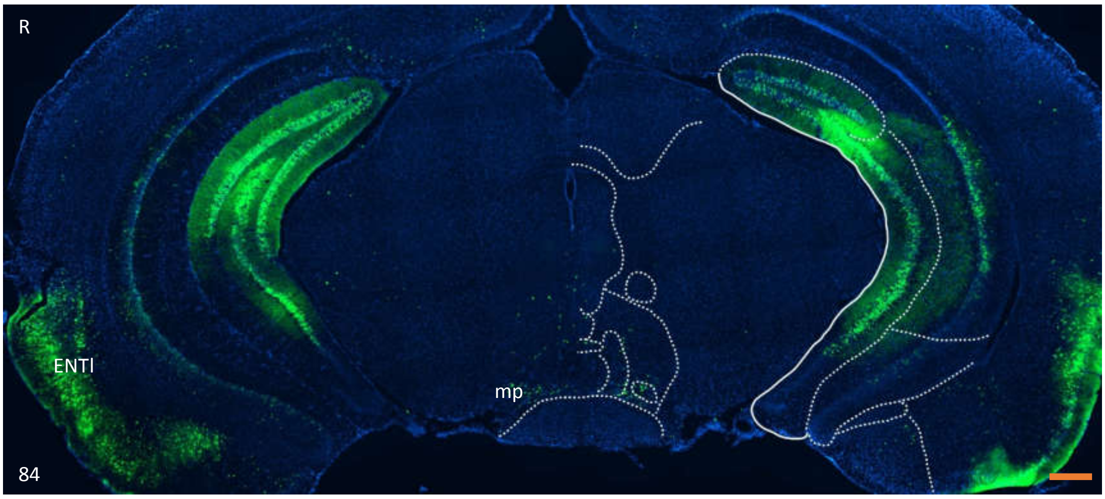

Biology Open • Supplementary information

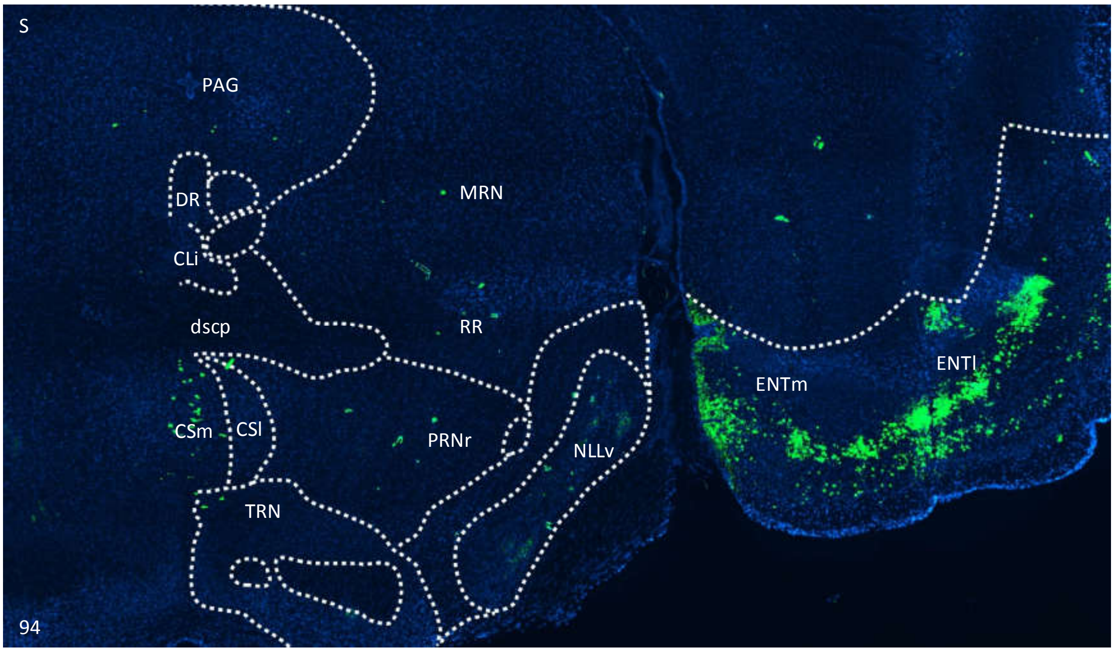

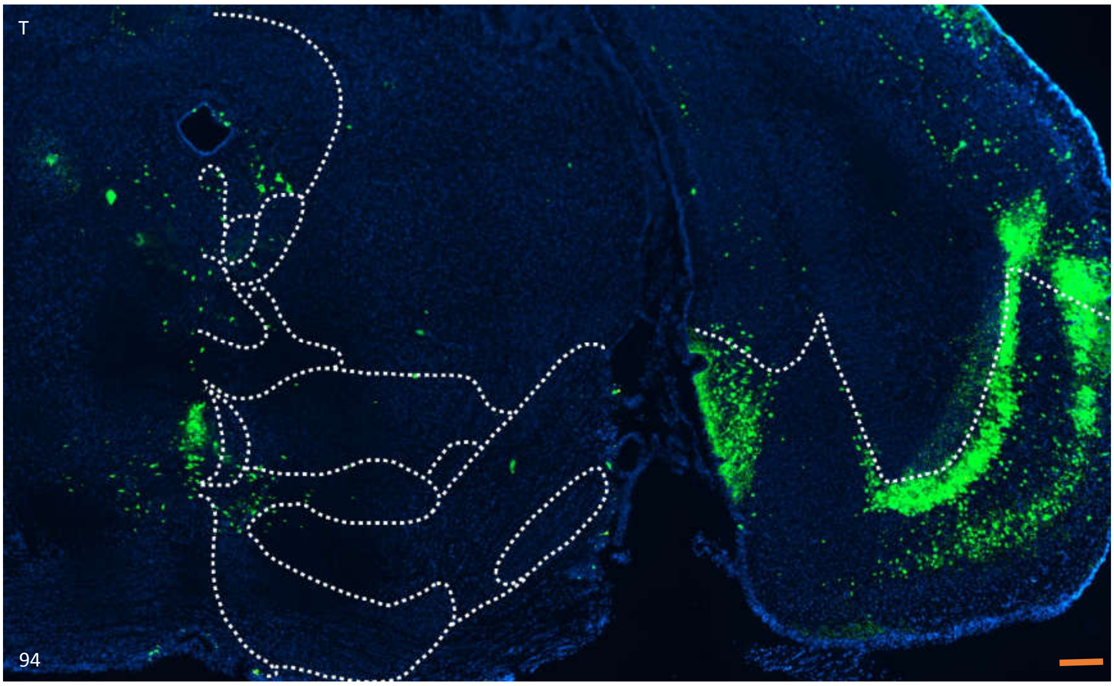

Biology Open • Supplementary information

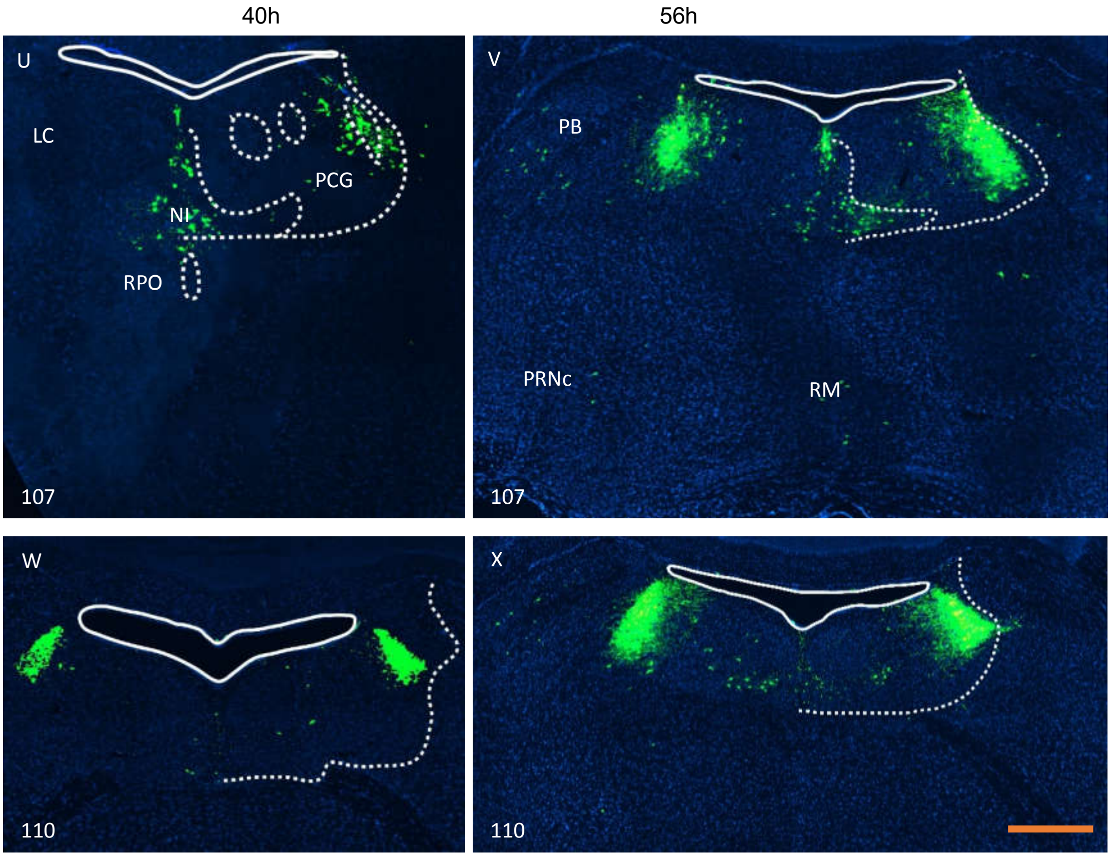

Fig. S3. The distribution of PRV-labeled axons at 40 and 56 hours after inoculation. The representative projections of virus PRV in the whole brain. A, C, E, G, I, K, M, O, Q, S, U, and W were shown projection at 40 hours after inoculation, B, D, F, H, J, L, N, P, R, T, V, X were shown projection at 56 hours after inoculation. Orange bars were present 200μm.

Abbreviations:

ACB: nucleus accumbens; ADP: anterodorsal preoptic nucleus; AHN: anterior hypothalamic nucleus; ATN: anterior group of the dorsal thalamus; BLA: basolateral amygdalar nucleus; BMA:basomedial amygdalar nucleus; BST: bed nuclei of the stria terminalis; CA1: field CA1; CA3: field CA3; CEA: central amydalar nucleus; CLi: central linear nucleus raphe; CM: central medial nucleus of the thalamus; CS: superior central nucleus raphe; CSl: lateral part of superior central nucleus raphe; CSm: median part of superior central nucleus raphe; DG: dentate gyrus; DMH: dorsomedial nucleus of the hypothalamus; DR: dorsal nucleus raphe; dscp: superior cerebellar peduncle decussation; DTN: dorsal tegmental nucleus; IMD: intermediodorsal

Biology Open • Supplementary information

nucleus of the thalamus; int: intimal capsule; LA: lateral amygdalar nucleus; LC: locus seruleus; LD: lateral dorsal nucelus of thalamus; LHA: lateral hypothalamic area; LHA: lateral hypothalamic area; LPO: lateral preoptic area; LSc: caudal part of the lateral septal nucleus; LSr: rostral part of lateral septal nucleus; dl: dorsolateral zone on LSr; m: medial zone on LSr; vl: ventrolateral zone on LSr; LSV: ventral part of the lateral septal nucleus; MEA: medial amygdalar nucleus; MEPO: median preoptic nucleus; MH: medial abenula; MM: medial mammillary nucleus, MPN: medial preoptic nucleus; MPO: medial preoptic area; MS: medial septal nucleus; MSC: medial septal nucleus complex; NI: nucleus incertus; PAG: periaqueductal gray; DR: dorsal nucleus raphe; PCG: pontine central gray; PH: posterior hypothalamic nucleus; smd: supramammillary decussation; PRNr: pontine reticular nucleus; PVH: paraventricular hypothalamic nucleus; Pvpo: preoptic part of the periventricular hypothalamic nucleus; PVT: paravntricular nucleus of the thalamus; RE: nueleus reuniens; RH: rhomboid nucleus; RE: nucleus of reuniens; RM: nucleus raphe magnus; RT: reticular nucleus of the thalamus; SF: septofimbrial nucleus; fi: fimbria; SH: septohippocampal nucleus; slm: stratum lacunosum-molecular; SMT: submedial nucleus of the thalamus; so: stratum oriens; sp: pyramidal layer; sr: srtratum rediatm; SUM: supramammillary nucleus; TRN: tegmental reticular nucleus; TRS: triangular nucleus of septum; VAL: ventral anterior-lateral complex of the thalamus; vl: ventrolateral zone on LSr; VMH: ventromedial nucleus of the hypothalamus; ZI: zona incerta.
